# Cognitive changes preceding Parkinson's disease: a systematic review and meta-analysis of prospective population-based studies

**DOI:** 10.3389/fnagi.2025.1627221

**Published:** 2025-10-07

**Authors:** Noelia Peña Arauzo, Christoph Theyer, Florian Krismer, Atbin Djamshidian, Werner Poewe, Corinne Horlings, Beatrice Heim, Laura Zamarian, Philipp Mahlknecht

**Affiliations:** Department of Neurology, Medical University of Innsbruck, Innsbruck, Austria

**Keywords:** cognition, prodromal, neuropsychology, executive function, non-motor symptoms

## Abstract

**Introduction:**

Various non-motor symptoms have been studied as part of the prodromal phase of Parkinson's disease (PD). However, studies assessing cognitive changes are scarce.

**Methods:**

We systematically searched PubMed and SCOPUS to identify prospective, population-based studies that reported on cognitive performance in individuals without PD at baseline, the incidence of PD at follow-up, and comparisons of cognitive performance between participants who developed PD and controls.

**Results:**

Twelve studies were identified, including a total of 524,807 participants, of whom 2,939 developed PD. Four studies found differences in global cognition and a meta-analysis showed that individuals who developed PD scored 0.3 points lower than controls on the Mini-Mental State Examination at baseline. Cognitive changes were most frequently observed in tests of executive function, processing-speed and attention, and less frequently in visuospatial/visuoconstructive skills and memory. Due to the heterogeneity of the assessment methods used, it was not possible to conduct further meta-analyses.

**Conclusions:**

Cognitive changes may be part of the clinical picture in prodromal PD cohorts derived from the general population. Further population-based studies with large samples and long-term follow-up are needed to better understand their extent and significance.

**Systematic review registration:**

https://www.crd.york.ac.uk/PROSPERO/view/CRD42024547252, identifier: CRD42024547252.

## 1 Introduction

The diagnosis of Parkinson's Disease (PD) is primarily based on the presence of cardinal motor features (Postuma et al., 2015), which develop due to degeneration of dopaminergic neurons in the substantia nigra. In addition, a variety of non-motors symptoms—caused by extranigral Lewy-body pathology and associated neuronal loss—also constitute an integral part of the disease, even in its earliest stages.

The prodromal phase of PD is mainly characterized by non-motor symptoms (NMS) such as hyposmia, constipation, REM sleep behavior disorder (RBD), and anxiety and/or depression (Mahlknecht et al., 2015). Except for RBD, these NMS are common in the general elderly population. Their association with incident PD has been extensively studied and is well established, leading to their inclusion in the Movement Disorder Society (MDS) research diagnostic criteria for prodromal PD (Berg et al., 2015). Among these NMS, isolated RBD has the highest specificity for future PD; in PD patients, the presence of RBD is associated with a more malignant phenotype, characterized by a higher burden of motor symptoms and NMS, including dysautonomia and cognitive impairment (De Pablo-Fernández et al., 2019).

Cognitive impairment is typically regarded as one of the principal NMS in the mid to late stages of PD and has been associated with reduced quality of life, loss of autonomy, and increased mortality (Foubert-Samier et al., 2020). Less is known about the types and incidence of cognitive abnormalities during the prodromal phase of PD, although cognitive changes have been reported to occur prior to diagnosis. Based on findings from three longitudinal studies (Darweesh et al., 2017a; Schrag et al., 2019; Weintraub et al., 2017), mild cognitive impairment (MCI) has been incorporated into the updated MDS criteria for prodromal PD (Heinzel et al., 2019). However, these studies are heterogeneous in many aspects, most notably in the assessment methods used and the baseline populations selected (enhanced risk vs. population-based).

In this study, we systematically reviewed prospective studies that report objective cognitive measures in individuals who were subsequently diagnosed with PD (i.e., individuals with presumed prodromal PD at baseline) to delineate the extent and range of cognitive changes occurring before the diagnosis of PD. We specifically focussed on studies conducted in population-based samples, as less is known in this setting, as opposed to studies in very high-risk cohorts or known prodromal populations, such as genetic mutation carriers or individuals with isolated RBD (Joza et al., 2023; Postuma et al., 2019). Furthermore, we conducted a meta-analysis to evaluate the association between lower baseline scores in a measure of global cognition and the incidence of PD.

## 2 Methods

The study was conducted in accordance with the Preferred Reporting Items for Systematic Reviews and Meta-Analyses (PRISMA) guidelines (Page et al., 2021) and was registered in PROSPERO (CRD42024547252).

### 2.1 Search strategy

We performed a comprehensive search in PubMed (Medline) and SCOPUS, covering the articles published from 1999 until May, 2024. The search utilized the following keywords: “Parkinson's Disease” OR “Parkinson”; AND “prodromal” OR “high risk cohort”; AND “cognition”, “executive function”, OR “cognitive impairment”. Additionally, the reference lists of the identified articles were checked for relevant studies. Rayyan QCRI program (Ouzzani et al., 2016) and Excel were used to screen and identify relevant studies.

Two researchers (N.P.A. and P.M.) determined the selection procedure. One researcher (N.P.A.) conducted the literature search and prepared the list of identified articles for screening. Based on the predefined inclusion and exclusion criteria (see below), two researchers (N.P.A. and C.T.) initially screened titles and abstracts. The full texts of potentially relevant articles were then reviewed for final inclusion or exclusion. Disagreements were resolved through discussion between the reviewers.

### 2.2 Inclusion and exclusion criteria

The predefined selection criteria were as follows:

- Inclusion criteria: (1) peer-reviewed original research articles, (2) published in English, (3) prospective study design, (4) population-based or cohort studies, and (5) assessment of one or more aspects of cognition at baseline in relation to a subsequent diagnosis of PD.
- Exclusion criteria: (1) studies involving cohorts of isolated RBD or PD gene mutation carriers, (2) duplicate publications, (3) diagnosis of PD or dementia at baseline, and (4) studies that assessed cognition but did not specify the cognitive assessment tools used.

### 2.3 Data extraction and key measurements

The following information was extracted: sample type and size, number of participants and incident PD cases, follow-up duration and time to diagnosis, tools used to measure global cognition and/or other cognitive domains (if assessed), and outcomes. This process was performed independently by three researchers (N.P.A., L.Z., and P.M.), with disagreements resolved by consensus.

Since some articles retained after the initial screening and application of the inclusion and exclusion criteria reported on “subjective cognitive complaints”, a dedicated section on this aspect was added subsequently to provide additional information to the reader. Two studies were included in this section, both of which are part of the main analysis (Foubert-Samier et al., 2020; Darweesh et al., 2017a).

### 2.4 Quality assessment and risk of bias

The quality and risk of bias for each included study was assessed using the “Quality In Prognosis Studies” (QUIPS) tool (Hayden et al., 2013). Studies were rated as “low”, “moderate”, or “high” risk of bias based on six different criteria, such as study participation, study attrition, or outcome measurement.

### 2.5 Statistical analysis

We evaluated the proportion of articles that examined global cognition or specific cognitive domains, noting whether significant results were reported. Based on these findings, we ranked the cognitive domains from most to least commonly impaired. Additionally, a meta-analysis was conducted on the studies reporting results on a common measure of global cognition, specifically the Mini Mental State Examination (MMSE; Foubert-Samier et al., 2020; Chastan et al., 2019; Darweesh et al., 2017b; Pausch et al., 2016).

The meta-analysis was conducted in R (version 4.3.2, The R Foundation for Statistical Computing) using the “metafor” package (Viechtbauer, 2010). The dataset included means and standard deviations of MMSE scores for both controls and individuals with incident PD, as well as sample sizes for each group. The standardized mean difference (SMD; Hedges, 1981) was calculated using the “escalc” function from the “metafor” package. A random-effects model was fitted with the “rma” function to account for variability across studies. Heterogeneity was assessed using τ^2^ (variance of heterogeneity), *I*^2^ (proportion of heterogeneity in total variability), and *H*^2^ (ratio of total variability to sampling variability).

## 3 Results

### 3.1 Study selection and risk of bias

**Figure 1:**
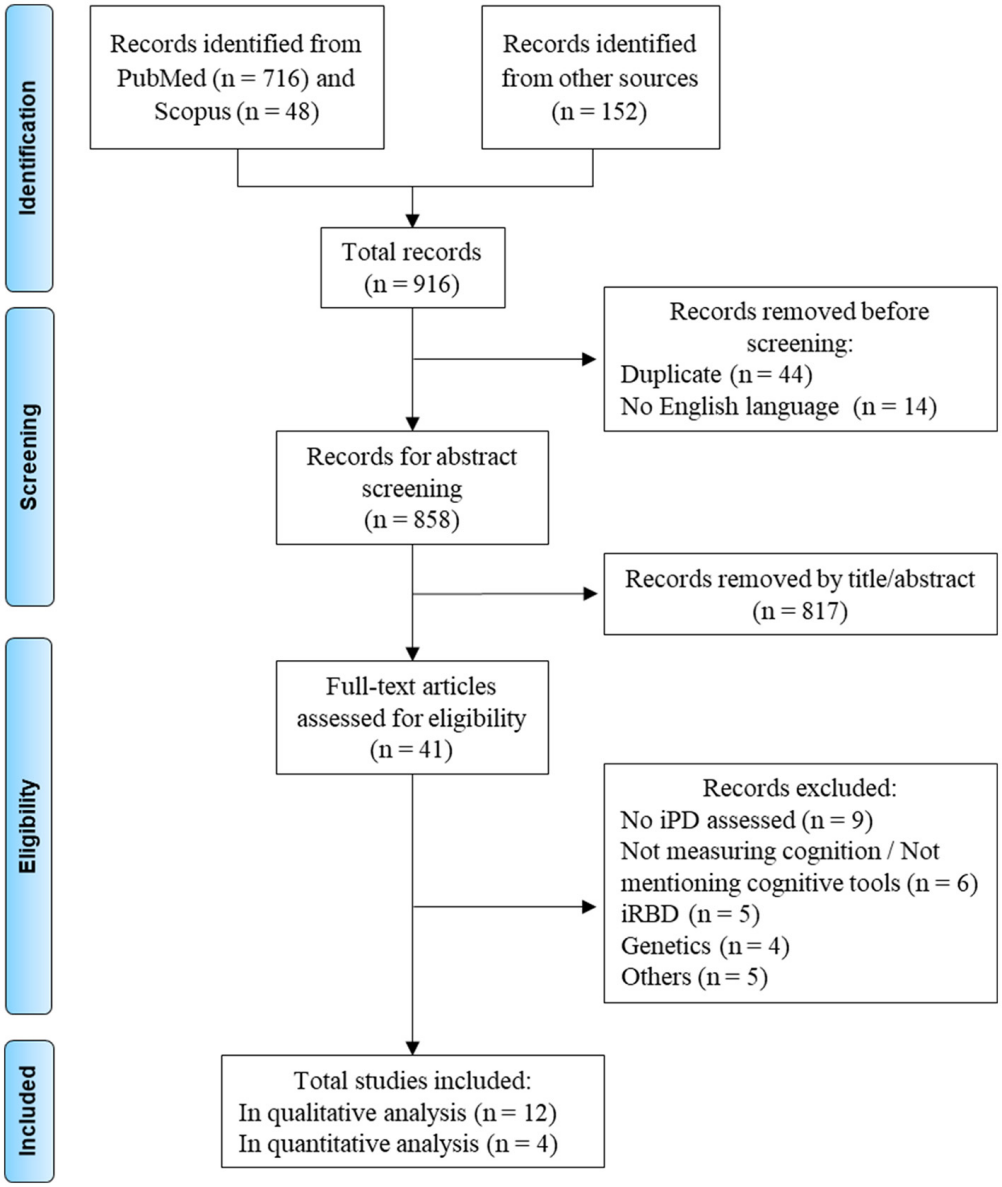
Flowchart of the different phases of the selection process. The category “Others” includes one review, three cross-sectional studies, and one study not directly related to Parkinson's Disease. iPD, idiopathic Parkinson's Disease; iRBD, idiopathic REM sleep behavior disorder.

**Figure 2:**
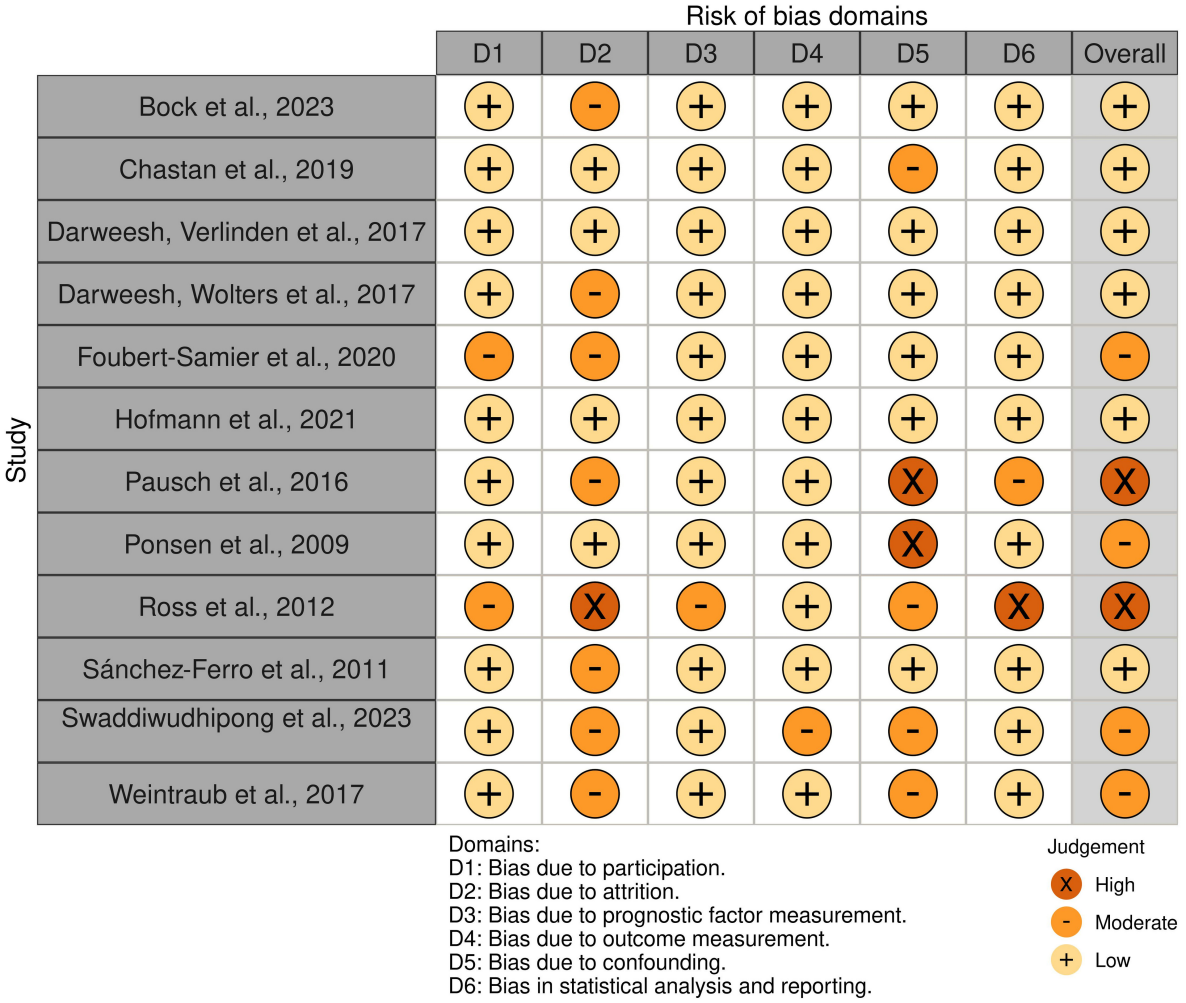
Traffic light plot of the risk of bias for the included studies.

**Figure 3:**
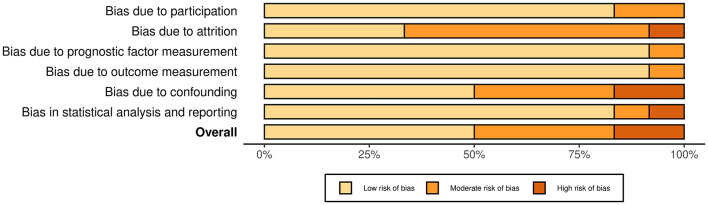
Summary plot of the risk of bias for the included studies.

The systematic literature review yielded 916 studies, which were screened for eligibility. After removing duplicates and non-English articles, and applying the inclusion and exclusion criteria, 12 studies were retained (Figure 1). The results of the risk of bias assessment are presented in Figures 2, 3.

### 3.2 Characteristics of the included studies

The characteristics of the 12 included studies (Foubert-Samier et al., 2020; Darweesh et al., 2017a,b; Weintraub et al., 2017; Chastan et al., 2019; Pausch et al., 2016; Hofmann et al., 2021; Bock et al., 2023; Ponsen et al., 2009; Ross et al., 2012; Swaddiwudhipong et al., 2023; Sánchez-Ferro et al., 2011), including the type of cognitive testing performed at baseline and its results according to incident PD (i.e. participants with a diagnosis of clinical PD at follow-up), are reported in Tables 1, 2. Notably, the study by Bock et al. (2023) analyzed male and female populations separately, resulting in two separate entries in Table 1. The study samples by Darweesh et al. (2017a,b) partially overlap; however, we treated them as independent studies because they used different global cognition scores and assessed an additional memory subdomain in one of the studies (Darweesh et al., 2017a).

**Table 1:** Prospective population-based studies assessing cognition that used incident PD as outcome.

**Study**	**Sample**	**N overall/N incident PD**	**FU time/time to diagnosis (years)**	**Global cognition**	**Domains assessed**	**Results**	**Comments**
Bock et al. (2023)	Prospective, population-based SOF	9,594 W/**129**	Up to 22 years/n.g.	MMSE (adapted version, scores 0-26)	EF	No significant results for global cognition Faster decline in EF in incident PD before diagnosis (but not after)	Significant sex differences were found in global cognition (at the time of diagnosis) and in EF (15 years before diagnosis). In both cases, scores were lower in men.
	Prospective, population-based MrOS	5,795 M/**168**	Up to 17 years/n.g.	3MS (scores 0-100)	EF	Faster decline in global cognition Faster decline in EF in incident PD before and after diagnosis	
Chastan et al. (2019)	Nested case control cohort, from the prospective, population-based BLSA	40/**10**	Mean 21 years/mean 2.6 years	MMSE	EF, LANG, MEM, PS/A, VSP	No differences in global cognition Differences in performance in EF and VSP	
Darweesh et al. (2017a)	Nested case control cohort, from the prospective, population-based Rotterdam study	1,199/**109**	Up to 23 years/up to mean 15.4 years	MMSE	EF, PS/A	Faster decline in global cognition in prediagnostic PD, separating from healthy controls around 5.6 years before diagnosis Faster decline in EF and PS/A in prediagnostic PD, separating from healthy controls between 7.1 and 3.3 years before diagnosis	Partly overlap with the cohort in Darweesh et al. (2017b) Assessment of subjective cognitive complaints included
Darweesh et al. (2017b)	Subcohort of the prospective, population-based Rotterdam study	7,386/**57**	Median 8.3 years/median 5.6 years	Global score derived from PCA of 4 tests, MMSE	EF, MEM, PS/A	No association of global cognition as defined by MMSE with probable PD Poor global cognition as defined by a global score from PCA of 4 tests associated with probable PD (HR 1.52; 95%CI 1.11-2.08) Poor performance in EF associated with probable PD (HR 1.56; 95%CI 1.20-2.00) Trend toward poorer performance in verbal fluency (HR 1.35; 95%CI 0.99-1.82) and memory recognition (HR 1.22; 95%CI 0.99-1.49)	Partly overlap with the cohort in Darweesh et al. (2017a) MMSE was assessed but not included in the main analysis.
Foubert-Samier et al. (2020)	Nested case control cohort, from the prospective population-based PAQUID study	129/**43**	Up to 15 years/mean 6.8 years	MMSE	EF, MEM, PS/A	No differences in global cognition Differences in PS/A (response times in ZCT) two years before diagnosis	Assessment of subjective cognitive complaints included
Hofmann et al. (2021)	Subcohort from the prospective, population-based TREND study	33/**12**	Up to 8 years/between 2 and 4 years	n.g.	EF, PS/A	No differences in EF and PS/A	
Pausch et al. (2016)	Subcohort (single center) of the prospective, population-based PRIPS	468/**5**	Mean 6.7 years/mean 5.4 years	MMSE, CERAD total score	EF, LANG, MEM, VSP	Poor global cognition as defined by MMSE (OR, 0.63; 95%CI 0.41–0.96) and CERAD total score (OR, 0.92; 95%CI 0.86-0.99) associated with PD conversion Poor performance in language (OR, 0.61; 95%CI 0.37-0.99) and memory (OR, 0.65; 95%CI 0.47-0.88) associated with PD conversion	Only 5 converters
Ponsen et al. (2009)	Prospective study in first-degree PD relatives	353/**5**	5 years/median 1.2 years	n.g.	EF	No significant results for EF	Only 5 converters
Ross et al. (2012)	Subcohort of the prospective, population-based HAAS	3,456 M/n.g.	8 years/n.g.	CASI total score	EF, LANG, MEM, PS/A, VSP	No significant results for global cognition Association of lower EF with higher PD incidence (unpublished data)	N incident cases for this subanalysis not given Results for domains other than EF not given
Sánchez-Ferro et al. (2011)	Nested case control, from the prospective, population-based NEDICES study	115/**23**	ca. 3 years/mean 1.3 years	37-MMSE total score	LANG, MEM, PS/A, VSP	No differences in global cognition No differences in cognitive subtests	Partly overlapping with Sánchez-Ferro et al., 2013
Swaddiwudhipong et al. (2023)	Prospective, population-based from the UK biobank	496,105/**2,370**	Mean 5–9 years/mean 7.4 years	n.g.	EF, MEM, PS/A	No significant results for any cognitive subtests	Not all participants performed all cognitive tests
Weintraub et al. (2017)	Prospective, population-based PARS	134/**8**	Mean 3.8 years/n.g.	Global score based on domain z-scores	EF, LANG, MEM, PS/A, VSP	Trend toward a significant association of lower global cognition (OR 2.6; 95%CI 0.9 - 7.1) with PD conversion Trend toward a significant association of lower EF (OR 2.7; 95%CI 1.0, 7.6) with PD conversion	Only 8 converters

3MS, Modified Mini-Mental State Examination; BLSA, Baltimore Longitudinal Study of Aging; CASI, Cognitive Abilities Screening Instrument; CERAD, Consortium to Establish a Registry for Alzheimer's disease; CI, confidence interval; EF, executive function; FU, follow-up; HAAS, Honolulu-Asia Aging Study; HR, hazard ratio; LANG, language; M, male; MEM, memory; MMSE, Mini Mental State Examination; MrOS, Osteoporotic Fractures in Men Study; N, number; n.g., not given; NEDICES, Neurological Disorders in Central Spain; OR, odds ratio; PAQUID, Personnes Agées Quid; PARS, Parkinson Associated Risk Syndrome; PCA, Principal Component Analysis; PD, Parkinson's disease; PRIPS, Prospective evaluation of Risk factors for Idiopathic Parkinson's Syndrome; PS/A, processing speed/attention; SOF, Study of Osteoporotic Fractures; TREND, Tübingen evaluation of Risk factors for Early detection of NeuroDegeneration; UK, United Kingdom; VSP, visuospatial/visuoconstructive abilities; W, women; ZCT, Zazzo's cancellation test. The number of participants who developed PD during the follow-up period is indicated in bold.

**Table 2:** Cognitive tests used in the different prospective population-based studies included in the review.

**Study**	**Global cognition**	**Executive function**	**Language**	**Memory**	**Processing speed/attention**	**Visuospatial abilities/visuoconstructive abilities**
Bock et al. (2023)	**MMSE (adapted version)/3MS**	**TMT-B**	n.g.	n.g.	n.g.	n.g.
Chastan et al. (2019)	MMSE	TMT-B, digit span backwards, **category fluency test**, letter fluency test	BNT	Immediate free recall, short-delay free recall, long-delay free recall (CVLT) BVRT	TMT-A, digit span forwards, DSST^Δ^	**Card rotation test**
Darweesh et al. (2017a)	**MMSE**	**Stroop test (Task 3), semantic fluency test**	n.g.	n.g.	**Stroop test (Task 1, Task 2), LDST**	n.g.
Darweesh et al. (2017b)	**Global score derived from PCA of 4 tests**, MMSE	Stroop test (Task 3)^*^, semantic fluency test^*^	n.g.	Word learning immediate task, word learning delayed task^*^, word learning recognition task	Stroop test (Task 1, Task 2), LDST^*^	n.g.
Foubert-Samier et al. (2020)	MMSE	Abstract thinking (WST), semantic fluency test (IST)	n.g.	BVRT	**ZCT**, DSST	n.g.
Hofmann et al. (2021)	n.g.	TMT-B (modified version)	n.g.	n.g.	TMT-A (modified version), TMT-C (control condition)	n.g.
Pausch et al. (2016)	**MMSE, CERAD total score**	Semantic fluency test (CERAD subtest)	**BNT (CERAD subtest)**	**Word list encoding**, word list recall, word list discriminability, constructional praxis recall (CERAD subtests)	n.g.	Constructional praxis copying (CERAD subtest)
Ponsen et al. (2009)	n.g.	Perseveration task, Corsi Block-Tapping Task (Vienna Test System subtests)	n.g.	n.g.	n.g.	n.g.
Ross et al. (2012)	CASI total score	**Semantic verbal fluency, judgement**, abstraction, digit span backwards, serial subtraction (CASI subtests)	Read and write, naming, following 3-step command (CASI subtests)	Orientation in time and space, recall of words and objects, date and place of birth, sunset hour (CASI subtests)	Repeating words and sentences (CASI subtests)	Copying pentagons (CASI subtest)
Sánchez-Ferro et al. (2011)	37-MMSE total score	n.g.	Language (37-MMSE subtest)	Orientation, immediate recall, delayed recall (37-MMSE subtests)	Attention and calculation (37-MMSE subtests)	Visuospatial copying (37-MMSE subtest)
Swaddiwudhipong et al. (2023)	n.g.	Reasoning test (fluid intelligence)	n.g.	Visual memory test (pairs-matching), prospective memory test, numeric memory test	Reaction time (snap game)	n.g.
Weintraub et al. (2017)	Global score based on domain z-scores	Phonemic fluency and semantic fluency (COWAT subtests), TMT- B, digit span backwards (WAIS-III subtest)	BNT	Immediate verbal recall, delayed verbal recall and recognition (HVLT-R), immediate story recall, delayed story recall and figural recall (RBANS subtests), logical memory story, immediate and delayed recall (WMS-III subtests)	Digit Symbol Coding, symbol search and digit span forward (WAIS-III subtests), TMT-A	Figure copying and line orientation (RBANS subtests), Clock Drawing Test, silhouettes (VOSP)

37-MMSE, Mini-Mental State Examination 37 items version; BNT, Boston Naming Test; BVRT, Benton Visual Retention Test; BVRT, Benton Visual Retention Test; CASI, Cognitive Abilities Screening Instrument; CERAD, Consortium to Establish a Registry for Alzheimer's disease; COWAT, Controlled Oral Word Association; CVLT, California Verbal Learning Test; DSST, Digit Symbol Substitution Test; HVLT-R, Hopkins Verbal Learning Test Revised; IST, Isaacs Set Test; LDST, Letter-Digit-Substitution Test; MMSE, Mini-Mental State Examination; n.g., not given; PCA, Principal Component Analysis; RBANS, Repeatable Battery for Assessment of Neuropsychological Status; TMT-A, Trail Making Test Version A; TMT-B, Trail Making Test Version B; TMT-C, Trail Making Test Control Version;VOSP, Visual object and Space Perception Battery; WAIS-III, Wechsler Adult Intelligence Scale Third Edition; WAIS-III, Wechsler Adult Intelligence Scale Third Edition; WMS-III, Wechsler Memory Scale Third Edition; WST, Wechsler Similarities test; ZCT, Zazzo's cancellation test.

The tests that showed significant group differences are indicated in bold.

^Δ^In case a test was classified into different categories, we decided to include it in the one that is more conventionally used (e.g., LDST in Darweesh et al., 2017b was classified as test for assessing EF and processing speed; we decided to include it in processing speed).

The PCA (Principal Component Analysis) of four tests is indicated by ^*^.

Sample sizes ranged from 33 to 496,105 participants, with a total of 2,939 individuals developing PD. Global cognition was evaluated in nine studies (Foubert-Samier et al., 2020; Darweesh et al., 2017a,b; Weintraub et al., 2017; Chastan et al., 2019; Pausch et al., 2016; Bock et al., 2023; Ross et al., 2012; Sánchez-Ferro et al., 2011). Across the 12 studies, various cognitive domains were assessed: executive function (reported in 11 studies; Foubert-Samier et al., 2020; Darweesh et al., 2017a,b; Weintraub et al., 2017; Chastan et al., 2019; Pausch et al., 2016; Hofmann et al., 2021; Bock et al., 2023; Ponsen et al., 2009; Ross et al., 2012; Swaddiwudhipong et al., 2023), memory (eight studies; Foubert-Samier et al., 2020; Weintraub et al., 2017; Chastan et al., 2019; Darweesh et al., 2017b; Pausch et al., 2016; Ross et al., 2012; Swaddiwudhipong et al., 2023; Sánchez-Ferro et al., 2011), processing speed and attention (nine studies; Foubert-Samier et al., 2020; Darweesh et al., 2017a,b; Weintraub et al., 2017; Chastan et al., 2019; Hofmann et al., 2021; Ross et al., 2012; Swaddiwudhipong et al., 2023; Sánchez-Ferro et al., 2011), visuospatial and visuoconstructive abilities (five studies; Weintraub et al., 2017; Chastan et al., 2019; Pausch et al., 2016; Ross et al., 2012; Sánchez-Ferro et al., 2011), and language (five studies; Weintraub et al., 2017; Chastan et al., 2019; Pausch et al., 2016; Ross et al., 2012; Sánchez-Ferro et al., 2011). The specific cognitive tests employed were highly heterogeneous; detailed descriptions are provided in Table 2. Below, we summarize the main findings, focusing on those studies reporting significant changes associated with incident PD. Supplementary Table 1 reports a list of the reviewed studies, highlighting the cognitive domains in which significant results were found.

#### 3.2.1 Global cognition

Nine studies assessed global cognition (Foubert-Samier et al., 2020; Darweesh et al., 2017a,b; Weintraub et al., 2017; Chastan et al., 2019; Pausch et al., 2016; Bock et al., 2023; Ross et al., 2012; Sánchez-Ferro et al., 2011), most commonly using the MMSE (Foubert-Samier et al., 2020; Darweesh et al., 2017a,b; Chastan et al., 2019; Pausch et al., 2016; Bock et al., 2023) or a modified version of it (Bock et al., 2023; Sánchez-Ferro et al., 2011). Differences in global cognition between participants who developed PD during follow-up and those who remained PD-free were identified in four studies (Darweesh et al., 2017a,b; Pausch et al., 2016; Bock et al., 2023), while five studies did not find such differences (Foubert-Samier et al., 2020; Weintraub et al., 2017; Chastan et al., 2019; Ross et al., 2012; Sánchez-Ferro et al., 2011).

Bock et al. (2023) used two samples—one from a male population-based study and one from a female population-based study—originally recruited for osteoporosis research. Follow-up assessments spanned up to 17 years in the male cohort and up to 22 years in the female cohort. Men with incident PD showed a greater decline in global cognition both prior to and following diagnosis, compared to men without incident PD. No differences were observed in the rates of change in global cognition among women with incident PD vs. those without. Additionally, men with incident PD scored significantly lower in global cognition at diagnosis than women with incident PD.

Two large prospective population-based studies within the Rotterdam Study (Darweesh et al., 2017a,b) also assessed global cognition. Darweesh et al. (2017b) derived a global cognition score via a Principal Component Analysis (PCA) of four measures (Stroop test—Part 3, letter-digit substitution test, verbal fluency test, and word learning delayed test) and observed that incident PD was associated with lower global scores. No association was found between MMSE scores and incident PD. Another nested case-control sample within the Rotterdam Study (Darweesh et al., 2017a) showed that MMSE scores declined more rapidly in incident PD cases than in controls, with trajectories of global cognition diverging approximately 5.6 years before diagnosis.

Finally, Pausch et al. (2016) compared the neuropsychological performance of individuals who developed PD over a 10-years period with that of controls. Significant differences were observed in global cognition measures (MMSE and the Consortium to Establish a Registry for Alzheimer's Disease [CERAD] total score), as well as in specific cognitive domains (see below); however, the study included only five incident PD cases.

#### 3.2.2 Executive function

Executive function was one of the most frequently investigated cognitive domains, assessed using various tests (see Table 2). Of the 11 studies evaluating this domain, five reported significant changes in incident PD cases (Darweesh et al., 2017a,b; Chastan et al., 2019; Bock et al., 2023; Ross et al., 2012), while six did not (Foubert-Samier et al., 2020; Weintraub et al., 2017; Pausch et al., 2016; Hofmann et al., 2021; Ponsen et al., 2009; Swaddiwudhipong et al., 2023).

Bock et al. (2023) identified significant differences in both samples. In the female cohort, incident PD individuals exhibited a faster decline in TMT-B performance compared to PD-free individuals prior to diagnosis, but not after. In contrast, differences in the male cohort between incident PD and PD-free participants persisted after diagnosis. Additionally, when comparing female and male subsamples with incident PD, men showed significantly lower executive function performance 15 years before diagnosis.

Chastan et al. (2019) evaluated executive function in a small nested case-control sample from the prospective, population-based Baltimore Longitudinal Study of Aging (BLSA), which follows human aging across the adult lifespan. They found lower scores on the category fluency test among incident PD cases relative to matched controls.

Darweesh et al. (2017a) reported significantly poorer scores on the Stroop test and semantic verbal fluency in incident PD cases compared to matched controls, with group differences emerging from 6.2 to 3.3 years before diagnosis. Similarly, Darweesh et al. (2017b) found that poor performance on the Stroop test is highly associated with probable PD.

The Honolulu-Asia Aging Study (Ross et al., 2012), involving over eight thousand Japanese-American men, investigated environmental, lifestyle, and physical characteristics, including various pre-motor features associated with PD. This study found that lower scores on Cognitive Abilities Screening Instrument (CASI) subtests assessing executive function (e.g., semantic verbal fluency, judgement) were associated with an increased likelihood of developing PD during an 8 years of follow-up period. However, this analysis was limited by low quality and high risk of bias within the study (see Figures 2, 3).

#### 3.2.3 Memory

Memory was also a frequently assessed cognitive domain in the included studies (Foubert-Samier et al., 2020; Weintraub et al., 2017; Chastan et al., 2019; Darweesh et al., 2017b; Pausch et al., 2016; Ross et al., 2012; Swaddiwudhipong et al., 2023; Sánchez-Ferro et al., 2011). The tests covered various aspects of verbal and figural, retrospective and prospective, as well as episodic and semantic memory (see Table 2). Of the eight studies, only one found significant baseline differences between PD converters and non-converters, specifically in verbal learning (Pausch et al., 2016). The remaining studies reported no significant differences in memory performance (Foubert-Samier et al., 2020; Weintraub et al., 2017; Chastan et al., 2019; Darweesh et al., 2017b; Ross et al., 2012; Swaddiwudhipong et al., 2023; Sánchez-Ferro et al., 2011).

#### 3.2.4 Processing speed and attention

Processing speed and attention were evaluated in nine studies (Foubert-Samier et al., 2020; Darweesh et al., 2017a,b; Weintraub et al., 2017; Chastan et al., 2019; Hofmann et al., 2021; Ross et al., 2012; Swaddiwudhipong et al., 2023; Sánchez-Ferro et al., 2011). Among these, significant differences between incident PD individuals and controls were observed in two studies (Foubert-Samier et al., 2020; Darweesh et al., 2017a). Notably, in the Rotterdam Study (Darweesh et al., 2017a), group differences emerged as early as 7.1 to 3.8 years before diagnosis, whereas in the prospective population-based *Personnes Agées Quid* (PAQUID) study (Foubert-Samier et al., 2020), changes were only detected 2 years prior.

#### 3.2.5 Visuospatial and visuoconstructive abilities

These abilities were assessed in five studies (Weintraub et al., 2017; Chastan et al., 2019; Pausch et al., 2016; Ross et al., 2012; Sánchez-Ferro et al., 2011), primarily through figure copying tests (Table 2). Overall, these assessments demonstrated limited sensitivity in detecting baseline changes among individuals with incident PD at follow-up. Notably, only one study identified significant differences between incident PD patients and controls using a mental rotation task (Chastan et al., 2019).

#### 3.2.6 Language

Language function was the least examined domain. Among the five studies (Weintraub et al., 2017; Chastan et al., 2019; Pausch et al., 2016; Ross et al., 2012; Sánchez-Ferro et al., 2011), only one found a significant difference in object naming performance between PD converters and controls (Pausch et al., 2016).

### 3.3 Meta-analysis

**Figure 4:**
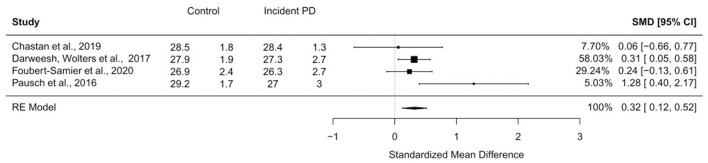
Forest plot of meta-analysis on global cognitive scores using the MMSE. Under the groups “Control” and “Incident PD”, the means and standard deviations of MMSE scores are listed. CI, confidence interval; RE model, random effect model; SMD, standardized mean difference.

Four studies (total *N* = 8023 individuals; 115 incident PD cases) (Foubert-Samier et al., 2020; Chastan et al., 2019; Darweesh et al., 2017b; Pausch et al., 2016) provided data suitable for meta-analysis on MMSE scores in incident PD participants. Results indicated that MMSE scores were significantly lower in those who developed PD during follow-up compared to PD-free individuals, with a standardized mean difference of 0.32 (Figure 4). The proportion of variability attributable to differences between the included trials as opposed to sampling error was low (*I*^2^ = 1%, *Q*[*df* = 3] = 5.23, *p* = 0.16).

### 3.4 Subjective cognitive complaints

Subjective cognitive complaints refer to self-perceived cognitive difficulties, which may encompass any cognitive domains (memory, executive function, processing speed, attention, language, and visuospatial abilities) (Jessen et al., 2014). It may or may not align with objective cognitive deficits.

Darweesh et al. (2017a) assessed memory complaints via three yes/no questions: (1) “Do you have more trouble remembering things than before?”; (2) “Does it happen more often that you are on your way to do something and forget what you wanted to do?”; and (3) “Do you more often have trouble finding words during a conversation?”. They found that differences in memory complaints between incident PD cases and controls appeared 1.5 years before diagnosis.

Foubert-Samier et al. (2020) assessed cognitive complaints through three questions about (1) “forgetfulness in daily living”; (2) “perceived difficulties in learning new information”; and (3) “perceived difficulties in word finding”, rated on a three-point Likert scale. Compared to controls, incident PD individuals reported significantly higher complaint scores between 12 and 4 years before diagnosis, but not thereafter. In the 4 years immediately prior, scores of incident PD cases were comparable to those of controls.

## 4 Discussion

This systematic literature review includes twelve prospective, population-based studies that examined objectively measured cognitive performance in individuals who were later diagnosed with PD—i.e. those presumed to be in the prodromal phase at baseline—compared to individuals who did not develop PD during follow-up (controls).

The findings are heterogeneous but collectively suggest that cognition may be mildly affected prior to diagnosis of PD. Of nine studies assessing global cognitive status, four found significant differences (Darweesh et al., 2017a,b; Pausch et al., 2016; Bock et al., 2023). This is supported by results of a meta-analysis showing that baseline MMSE scores were, on average, 0.3 points lower in future PD cases than in controls. Additionally, impairments appeared more frequently in tests of executive function and processing speed and attention, whereas deficits in visuospatial/visuoconstructive abilities and memory were less frequently observed. These domain-specific findings are substantiated by a recent comprehensive meta-analysis that also included cohort studies, such as those involving isolated RBD patients (Speelberg et al., 2022). This analysis found that poor executive function was associated with an increased risk of developing PD; in contrast, global cognitive impairment was not (Speelberg et al., 2022).

Several factors may explain the mixed results. First and most importantly, the included studies vary considerably in study populations and cognitive assessments. For example, in the Rotterdam study (Darweesh et al., 2017b), 7,386 participants were evaluated using nine tests covering global cognition, executive function, memory, processing speed, and attention. In contrast, the NEDICES study (Sánchez-Ferro et al., 2011) involved 115 participants assessed with the 37-item MMSE version. Second, cognitive changes may affect only a small proportion of individuals during the prodromal phase, similar to other prodromal NMS such as depression and anxiety (Jacob et al., 2010). Third, in affected individuals, cognitive changes may be subtle and therefore difficult to detect with the tests employed. Finally, different cognitive domains may be variably affected in individuals with incident PD, reflecting the heterogeneity observed in established PD.

The “dual syndrome” hypothesis postulates two broad profiles of cognitive impairment: (1) fronto-striatal dysfunction related to dopaminergic pathways degeneration, leading to impairments in executive function, working memory, and attention; and (2) posterior cortico-temporal dysfunction involving cholinergic pathways, resulting in visuospatial and recognition memory impairments (Brandão et al., 2020; Kehagia et al., 2013). According to this model, individuals with PD may develop or exhibit specific cognitive impairments depending on their patterns of pathology progression. In this literature review, executive function emerged as the most commonly impaired cognitive domain, consistent with experimental evidence suggesting earlier dopaminergic, rather than cholinergic, dysfunction in prodromal PD (Arnaldi et al., 2012; Hirano, 2021).

While most studies did not assess sex differences, one study focused on sex-specific cognitive trajectories before and after PD diagnosis (Bock et al., 2023). Women with incident PD showed no global cognitive decline, but showed a decline in executive function before diagnosis. Conversely, men with incident PD showed faster decline in global cognition before diagnosis and a prominent decline in executive function compared to women (Bock et al., 2023). This difference in global cognition may indicate some degree of sex-specific cognitive heterogeneity in the prodromal phase of PD. The executive function impairment observed only in men after diagnosis may be explained by the neuroprotective and neurotrophic effects of estrogen in women, which can also increase nigrostriatal dopaminergic activity (Bock et al., 2023; Bourque et al., 2019). It has been shown that women with higher lifetime exposure to estrogen have a lower risk of developing PD (Bourque et al., 2019), while those with lower lifetime estrogen exposure—such as earlier menarche (before age 12), later menarche (at age 14 or older), or a higher number of births—are associated with an increased PD risk.

Two of the twelve included studies also examined subjective cognitive complaints (Foubert-Samier et al., 2020; Darweesh et al., 2017a). Subjective cognitive complaints are known to carry a high risk of progression to neurodegenerative diseases, especially dementia. For instance, one longitudinal study assessed older participants with intact cognition or MCI at baseline, categorizing them based on the presence of self- or informant-reported complaints (Gifford et al., 2014). Among cognitively intact participants, those with any complaints had an increased risk of developing MCI or dementia. In MCI participants, only those with informant or combined (self and informant) complaints showed a higher risk of progression to dementia. Similarly, subjective motor complaints—such as stiffness or trembling—have been shown to increase the risk of future PD (De Lau et al., 2006; Schrag et al., 2015). Given the growing interest in cognitive impairment in PD, research has increasingly focussed on subjective cognitive complaints (Siciliano et al., 2024). Among the studies included in this review, two investigated this issue: the Rotterdam study (Darweesh et al., 2017a) found that subjective cognitive complaints appeared approximately 1.5 years before diagnosis, while the PAQUID study (Foubert-Samier et al., 2020) detected group differences between 12 and 4 years prior to diagnosis, but not after. The authors attributed these findings to depression, which can influence the individuals' perceptions about memory or cognition during PD development. Another cross-sectional study by Flores-Torres et al. (2021) reported more subjective cognitive complaints in individuals at high risk of developing PD (i.e. with one or more NMS) than in individuals without any prodromal PD features. A prospective six-years study by Schrag et al. (2023) found an association between subjective memory complaints (reported to general practitioners and recorded in a primary care database) and incident PD, although specific assessment tools or questions were not detailed. Taken together, these results highlight the potential of self-perceived cognitive difficulties as an early marker not only of AD, but of PD too. A recent systematic review and meta-analysis indicated a weak association between subjective cognitive complaints and objective cognitive performance in PD patients; however, patients who are cognitively healthy at baseline exhibit an increased risk of subsequent cognitive decline over an average follow-up of 3.16 years (Siciliano et al., 2024). In general, the predictive value of subjective cognitive complaints in neurodegenerative disorders remains under active investigation. While some studies suggest a link between early subjective complaints and future cognitive deterioration, others find no such association (Pitti et al., 2022; Reid and MacLullich, 2006), underscoring the need for further research. Notably, subjective cognitive complaints can also be assessed remotely, making them promising candidates for inclusion in cost-effective, internet-based screening batteries. Additionally, advances in device-based cognitive assessments, such as eye-tracking, may offer promising avenues for detecting changes related to neurodegenerative disorders while minimizing motor-related confounds (Bueno et al., 2019).

We should acknowledge limitations of the present literature review. First, there was considerable heterogeneity in the cognitive tests used (e.g., five different tests assessed language). Additionally, many studies relied on complex cognitive tests that simultaneously assess multiple domains, potentially obscuring domain-specific deficits. Second, the cognitive domains examined were unevenly represented, with most studies focussing on executive function, processing speed, attention, and memory, while fewer assessed language and visuospatial abilities. Third, some of the studies included had a substantial risk of bias. Fourth, there was a partial overlap between samples in two studies (Darweesh et al., 2017a,b). Due to the lack of precise information about this overlap, the final number of incident PD patients may not be completely accurate. Furthermore, the studies included did not specify whether participants had a genetic predisposition to develop PD or dementia. Fifth, most studies used the MMSE to assess global cognition, likely due to their population-based design and primary focus beyond neurodegenerative diseases. Despite the MMSE's broad screening nature, a statistically significant difference was observed in baseline MMSE scores between individuals with incident PD and controls. While a 0.3-point difference on the MMSE may not be clinically meaningful for single individuals, at a group level it provides valuable insights into early cognitive changes during the prodromal phase of PD. Specifically, these findings suggest that cognitive decline—detectable even through brief global assessments like the MMSE—may begin very subtly before more overt symptoms appear. In this context, more sensitive screening tools, such as the Montreal Cognitive Assessment (MoCA) or the Parkinson's Disease Cognitive Rating Scale (PD-CRS), may offer greater efficiency in detecting subtle cognitive changes in prodromal PD (Brandão et al., 2023; Yang et al., 2023). According to a recent review (Bezdicek et al., 2025), a comprehensive neuropsychological assessment—including tests of memory, visuospatial functions, and language—is recommended for the early identification of cognitive alterations in association with PD. Finally, our review primarily focused on objective cognitive measures. A section dedicated to subjective cognitive complaints was added retrospectively after noticing that two of the studies included also addressed this aspect. It is important to note that “subjective cognitive complaints” is just one of several terms used to describe this issue. Future research should consider the variability of terminology to facilitate a more comprehensive understanding of the significance of subjective cognitive changes in the prodromal phase of PD.

In conclusion, cognitive impairment may be present during the prodromal phase of PD. A comprehensive neuropsychological evaluation targeting the most affected cognitive domains, along with subjective cognitive complaints, could enhance early detection. Further prospective studies are needed to delineate the trajectory of cognitive changes in prodromal PD and determine their significance for risk screening and the subsequent clinical course of individual patients.

## Data Availability

The original contributions presented in the study are included in the article/Supplementary material, further inquiries can be directed to the corresponding authors.
