# Supplementary material for: Cognitive changes preceding Parkinson's disease: a systematic review and meta-analysis of prospective population-based studies
Source: Front Aging Neurosci. 2025 Oct 7;17:1627221. doi: 10.3389/fnagi.2025.1627221 (PMC12537721; doi:10.3389/fnagi.2025.1627221)
Supplement: Supplementary file 1 [file Data_Sheet_1.pdf]

**Supplementary Table 1:** List of studies indicating the cognitive domains assessed and their significant results.

| | GLOBAL COGNITION | EXECUTIVE FUNCTION | LANGUAGE | MEMORY | PROCESSING SPEED / ATTENTION | VISUOSPATIAL ABILITIES |
|---|---|---|---|---|---|---|
| **Bock et al., 2023** | **1** | **1** | - | - | - | - |
| **Chastan et al., 2019** | 0 | **1** | 0 | 0 | 0 | **1** |
| **Darweesh, Verlinden et al. 2017** | **1** | **1** | - | - | **1** | - |
| **Darweesh, Wolters et al., 2017** | **1** | **1** | - | 0 | 0 | - |
| **Foubert-Samier et al., 2020** | 0 | 0 | - | 0 | **1** | - |
| **Hofmann et al., 2021** | - | 0 | - | - | 0 | - |
| **Pausch et al., 2016** | **1** | 0 | **1** | **1** | - | 0 |
| **Ponsen et al., 2009** | - | 0 | - | - | - | - |
| **Ross et al., 2012** | 0 | **1** | 0 | 0 | 0 | 0 |
| **Sánchez-Ferro et al., 2011** | 0 | - | 0 | 0 | 0 | 0 |
| **Swaddiwudhipong et al., 2023** | - | 0 | - | 0 | 0 | - |
| **Weintraub et al., 2017** | 0 | 0 | 0 | 0 | 0 | 0 |
| | | | | | | |
| **N of studies assessing a domain** | 9 | 11 | 5 | 8 | 9 | 5 |
| **N of studies showing significant differences in a specific domain** | 4 | 5 | 1 | 1 | 2 | 1 |
| **Proportion of studies assessing a domain** | 75% | 92% | 41% | 67% | 75% | 41% |
| **Proportion of studies showing significant differences in a specific domain** | 44% | 45% | 20% | 13% | 22% | 20% |

**1**: significant differences; 0: no significant differences; -: not applicable.
